# *CYFIP1* overexpression amplifies IL-6/STAT3 and IFN-γ/STAT1 signaling: potential implications for neuroinflammation and autism spectrum disorder

**DOI:** 10.1016/j.bbih.2026.101293

**Published:** 2026-06-22

**Authors:** Emily-Rose Martin, Josan G. Martin, Mark A. Russell, Asami Oguro-Ando

**Affiliations:** a University of Exeter Medical School, University of Exeter, Exeter, United Kingdom; b Faculty of Pharmaceutical Sciences, Tokyo University of Science, Tokyo, Japan

**Keywords:** Autism spectrum disorder, CYFIP1, JAKMIP1, Cytokine signaling, IL-6, STAT3

## Abstract

**Background and objectives:**

Autism spectrum disorder (ASD) encompasses a group of neurodevelopmental disorders influenced by genetic and environmental factors, although the molecular mechanisms underlying their interactions remain unclear. We previously reported dysregulated cytokine signalling in chromosome 15q-duplication syndrome (Dup(15q)), a common syndromic form of ASD. Dup(15q) induced pluripotent stem cell (iPSC)-derived neurons exhibit an amplified Signal Transducer and Activator of Transcription-3 (STAT3) response to Interleukin-6 (IL-6), a cytokine often upregulated in ASD. To identify candidate genes within the 15q region that may modify cytokine signalling, we investigated *Cytoplasmic FMRP-Interacting Protein 1* (*CYFIP1*), as *CYFIP1* dysregulation has been linked to altered expression of genes involved in immunoregulatory pathways.

**Methods:**

*CYFIP1* was overexpressed using a plasmid vector in human HEK-293 and SH-SY5Y neuroblastoma cells. Following stimulation with IL-6 or Interferon (IFN)-γ, a variety of biochemical assays (qRT-PCR, Western blotting and dual-luciferase reporter assays) and neurite tracing experiments were performed to assess the effects of increased *CYFIP1* expression on IL-6/STAT3 and IFN-γ/STAT1 signaling responses.

**Results:**

*CYFIP1*-overexpression HEK-293 cells display reduced *STAT3* and *STAT1* expression, but enhanced IL-6-induced/IFN-γ-induced STAT3/STAT1 transcriptional activity. Furthermore, *CYFIP1*-overexpression in SH-SY5Y cells was associated with reduced basal neurite outgrowth and altered IL-6-associated neurite outgrowth.

**Conclusions:**

These findings suggest that *CYFIP1*-overexpression modifies cytokine-responsive transcriptional pathways in vitro and provides novel insight into how *CYFIP1* dysregulation may contribute to dysregulated cytokine signaling in ASD, advancing our understanding of the molecular mechanisms underlying neuroinflammatory processes in this disorder.

## Highlights

• *CYFIP1* -overexpression reduced the expression of *STAT1* and *STAT3* in HEK-293 cells.
• Despite this, increased expression of *CYFIP1* in HEK-293 cells enhances IL-6-induced STAT3 and IFN-γ-induced STAT1 activity.
• *CYFIP1*-overexpressing SH-SY5Y cells show altered IL-6-associated neurite outgrowth.

## 1 Background

Autism spectrum disorder (ASD) is a heterogeneous group of neurodevelopmental conditions in which both genetic variation and environmental exposures contribute to pathogenesis (American Psychiatric Publishing, 2016). Due to the highly heterogeneous nature of the disorder, both diagnosis and treatment remain challenging. Genetic factors, including both rare and common variants, are implicated in ASD, with over 1000 genes identified as potentially contributing to its onset and development (Abrahams et al., 2013). ASD heritability is estimated to be between 26 and 93% (Bourgeron, 2015), with concordance rates between siblings around 30%, dizygotic twins about 65%, and up to 99% in monozygotic twins (Ramaswami and Geschwind, 2018; Colvert et al., 2015; Hallmayer et al., 2011; Rosenberg et al., 2009; Bailey et al., 1995). In addition to genetic predisposition, environmental risk factors, particularly maternal immune activation (MIA) and neuroinflammation, have been recognised as key contributors to ASD pathogenesis (Modabbernia et al., 2017; Zawadzka et al., 2021). Therefore, it is likely that the interaction between an individual's genetic background and specific environmental exposures plays a crucial role in triggering the pathogenesis underlying ASD (Modabbernia et al., 2017). Consequently, elucidating the molecular mechanisms by which ASD-associated genes can alter neurodevelopmental processes in response to environmental stressors is critical for advancing our understanding of the disorder.

*Janus Kinase and Microtubule-Interacting Protein 1* (*JAKMIP1*) was previously identified as a potential molecular link between syndromic forms of ASD and neuroinflammation (Nishimura et al., 2007). *JAKMIP1* encodes a multi-functional protein that possesses microtubule- and RNA-binding capabilities, associates with Type B GABA Receptors and Janus Kinases (JAKs), and is reported to be involved in various cellular processes, including microtubule stabilisation, intracellular trafficking, neurite outgrowth, neuronal migration and local activity-dependent protein translation (Steindler et al., 2004a; Vidal et al., 2007, 2012; Berg et al., 2015; Couve et al., 2004). *JAKMIP1* expression is commonly dysregulated in Fragile X syndrome (FXS) and chromosome 15q-duplication syndrome (Dup(15q)), both genetic disorders often comorbid with ASD (Nishimura et al., 2007). Furthermore, we discovered a role for JAKMIP1 in regulating Interleukin-6 (IL-6)-induced Signal Transducer and Activator of Transcription-3 (STAT3) signaling in neuronal cells, whereby deficiency of JAKMIP1 in SH-SY5Y neuroblastoma cells impairs STAT3 activation and neuritogenesis in response to IL-6 stimulation (Martin et al., 2025). These findings suggest that dysregulation of *JAKMIP1* in ASD may alter the ability of neurons to respond to inflammatory cytokines, potentially leading to aberrations in neuronal morphology.

To investigate whether JAKMIP1-regulated IL-6/STAT3 signaling was relevant to syndromic forms of ASD, we previously assessed IL-6-induced STAT3 signaling in a Dup (15q) induced pluripotent stem cell (iPSC)-derived neuronal model (Martin et al., 2025). We discovered that whilst *JAKMIP1* expression fluctuated in Dup(15q) iPSCs and iPSC-derived cortical neurons, IL-6-induced STAT3 activity was significantly enhanced in both Dup(15q) iPSCs and iNeurons (Martin et al., 2025). These findings suggested that some gene (or collection of genes) in the q arm of chromosome 15 likely exerts stronger influence on STAT3 activity in the context of ASD.

Several genes are encoded within the 15q region of chromosome 15, however, a gene of particular interest in relation to ASD and cytokine signaling is *Cytoplasmic FMRP-Interacting Protein 1* (*CYFIP1*), as studies have demonstrated that duplications (or even deletions) encompassing *CYFIP1* increase the severity of neurobehavioural phenotypes (Butler et al., 2004), and that *CYFIP1* expression is upregulated in the brains of individuals with Dup(15q) and ASD (Oguro-Ando et al., 2015). Although CYFIP1 is most known for its roles in repressing neuronal protein translation as part of Fragile X Messenger Ribonucleoprotein (FMRP)-containing ribonucleoprotein complexes (Napoli et al., 2008) and regulating actin polymerisation as a core component of the WAVE regulatory complex (WRC) (DeRubeis et al., 2013), there is evidence to suggest that *CYFIP1* dysregulation may alter the expression of various genes involved in immunoregulatory signaling pathways (Urraca et al., 2018). For example, a recent study profiling transcriptomic changes in Dup(15q) stem cell-derived neurons with upregulated *CYFIP1* expression suggested that increased dosage of chromosome 15q11.2 genes could alter the expression of gene targets of SMAD Family Member 3 (SMAD3), Nuclear Factor Kappa B (NF-κB) or Interferons (IFN) (Urraca et al., 2018). Moreover, another study identified *CYFIP1* as a potential hub gene that may coordinate Transforming Growth Factor Beta 1 (TGF-β1)/SMAD3 signaling (Erickson et al., 2019). Therefore, we hypothesised that CYFIP1 may be responsible for the observed enhanced IL-6-induced activity in Dup (15q) iPSCs and iNeurons (Martin et al., 2025).

This work explores the effects of increased *CYFIP1* expression (modelled by *CYFIP1*-overexpression (OE)) on cytokine signaling. We provide the first evidence to directly support a novel role for CYFIP1 in modulating cytokine signaling, demonstrating that *CYFIP1*-OE in Human embryonic kidney (HEK)-293 cells alters the expression and activity of cytokine signaling-related transcription factors, *STAT1* and *STAT3*. Taking this further, we show how alterations in the STAT3 signaling pathway due to *CYFIP1*-OE affects IL-6-induced neurite outgrowth in human SH-SY5Y neuroblastoma cells, illustrating that these findings continue to be relevant in a more neuronal-relevant model.

## 2 Methods

Full methodology details can be found in Supplementary File 1.

### 2.1 HEK-293 and SH-SY5Y cell culture

HEK-293 cells (#CRL-1573™, *American Type Culture Collection*) and human neuroblastoma SH-SY5Y cells (#CRL-2266™, *American Type Culture Collection*) were maintained in Dulbecco's modified eagle medium/nutrient mixture F-12 with GlutaMAX (referred to henceforth as DMEM/F-12) (#11524436, *Fisher Scientific*™) supplemented with 10% foetal bovine serum (FBS) (#11550356, *Fisher Scientific*™), and incubated at 37°C, 5% CO_2_, 95% humidity. Cells were passaged at 70-80% confluency using TrypLE™ (#10718463, *Fisher Scientific*™).

### 2.2 Cytokine treatments

Cells were stimulated with 20 ng/mL IL-6 (#7270IL-025, *R&D Systems*) or 20 ng/mL Interferon-γ (IFN-γ) (#285-IF-100, *R&D Systems*) diluted in DMEM/F-12 + FBS (or in DMEM/F-12 supplemented with Retinoic Acid (RA) and Brain-Derived Neurotrophic Factor (BDNF) for neurite tracing experiments). These cytokine concentrations and time-points were chosen as we have previously found them to be sufficient for inducing a STAT3 or STAT1 response (Martin et al., 2025; Dhayal et al., 2022). For all experiments using IL-6 or IFN-γ, control cells were used which were treated with corresponding media not containing IL-6 or IFN-γ. A schematic overview of the cytokine treatment conditions and time-points can be found in Supplementary Figure 1.

### 2.3 Plasmid transfection

For signalling pathway experiments, HEK-293 cells were transfected with either an RFP-T2A-CYFIP1 plasmid to induce *CYFIP1*-overexpression (these cells are referred to as ‘*CYFIP1*-OE cells'), or the backbone RFP-T2A plasmid as a control (these cells are referred to as ‘control cells’) using Lipofectamine™ LTX Reagent (#15338100, *Fisher Scientific*™), following manufacturer instructions. Media was replaced the following day following transfection and all experiments were performed 72 h post-transfection.

For neurite tracing experiments, SH-SY5Y cells were transfected by Nucleofection™ (#V4XC-2012, *Lonza Bioscience*), following manufacturer instructions, with either a mixture of the RFP-T2A backbone vector and pCAβ-YFP (encoding yellow fluorescent protein (YFP)) in a 1:1 ratio as the control condition; or a mixture of RFP-T2A-CYFIP1 and pCAβ-YFP (4:1 ratio) to assess the effects of *CYFIP1*-OE on neuritogenesis. The pCAβ-YFP plasmid was co-transfected to enable better visualisation of the cells for neurite tracing as the RFP encoded by the *CYFIP1*-OE plasmid photo-bleaches quickly. Media was replaced the following day after Nucleofection™.

### 2.4 Protein extraction and Western blotting

Cells were harvested in ice-cold RIPA lysis buffer supplemented with protease and phosphatase inhibitors. 50 μg of total protein was heated in Laemmli buffer for 5 min at 95°C and loaded into 4–15% precast polyacrylamide gels (#4561085, *Bio-Rad Laboratories*) for Western blotting alongside a pre-stained protein ladder (#PL00001, *Proteintech Group*). Following electrophoresis, proteins were transferred onto methanol-activated PVDF membranes (#IPVH304F0, *Millipore*), then sequentially incubated in blocking buffer and antibody solutions. Proteins were visualised using the Odyssey® CLx Imager (*LI-COR Biosciences*) and the Image Studio software (Version 5.2; *LI-COR Biosciences*) was used to perform densitometric analyses to compare the abundance of target proteins between samples. Full uncropped Western blot images for each primary antibody used can be found in Supplementary Figures 2-4, and antibody details and dilutions can be found in Supplementary File 1: Table 1.

### 2.5 RNA extraction, cDNA conversion and qRT-PCR

Total RNA was isolated and purified using the Direct-zol™ RNA Miniprep kit following manufacturer instructions (#R2051, *Zymo Research*). RNA was converted to cDNA using PrimeScript™ RT reagent kit (#RR037A, *Takara Bio Europe*) following manufacturer instructions. qRT-PCR was then performed using HOT FIREPol® EvaGreen® qPCR Master Mix with ROX (#01-02-00500, *Solis BioDyne*) with the QuantStudio 12K Flex qPCR machine (*Thermo Fisher Scientific*™). For primer sequences, see Supplementary File 1: Table 2. Each reaction was run in triplicate, and mRNA expression levels were normalised against the geometric mean of two reference genes using the Pfaffl method (Pfaffl, 2001). Gene expression ratios were then presented in figures relative to the control cells.

### 2.6 Dual-luciferase reporter assays

HEK-293 cells were transfected with STAT3 Cignal Reporter plasmid (#336841, GeneGlobe ID: CCS-9028L, *Qiagen*) or Gamma-ativated sequence (GAS) Cignal Reporter plasmid (#336841, GeneGlobe ID: CCS-009L, *Qiagen*) using Lipofectamine™ LTX Reagent. Six hours post-transfection, cells were treated with DMEM/F-12 + 10% FBS with or without 20 ng/mL IL-6 or IFN-γ and incubated for 18 h prior to the reporter assay. Firefly and Renilla luciferase activities were measured using the Dual-Luciferase® Reporter (DLR™) Assay System (#E1960, *Promega*), following manufacturer instructions, with the PHERAstar FS microplate reader (*BMG LABTECH*).

### 2.7 SH-SY5Y differentiation and neurite outgrowth analysis

SH-SY5Y cells were seeded onto sterile glass coverslips (#631-1578, *VWR*®; pre-coated with 20 μg/mL poly-D-Lysine (PDL; #354210, *Corning*®)) in DMEM/F-12 + 10% FBS. The following day, media was replaced with DMEM/F-12 (without FBS) containing 20 ng/mL BDNF and 10 μM RA. 48 h later, SH-SY5Y cells were fixed with 4% paraformaldehyde (PFA), and nuclei were counterstained with 4′,6-diamidino-2-phenylindole (DAPI) (#D9542, *Sigma-Aldrich*®) prior to coverslip mounting onto microscope slides. Slides were imaged on the inverted DMi8 widefield microscope (*Leica Microsystems*).

All image analysis was performed manually using the *FIJI* software (*ImageJ*; Version 2.9.0 (Schindelin et al., 2012)). The length of the longest neurite produced by a single cell (referred to as longest neurite length (LNL); a measure of neurite extension) and the sum length of all neurites produced by a single cell (referred to as total neurite length (TNL); a measure of neuritogenesis) were measured.

### 2.8 Statistical analysis

Statistical analyses were performed using GraphPad Prism (GraphPad Prism Version 9.0.0 for Windows, GraphPad Software, San Diego, California USA, www.graphpad.com). Unpaired Student's t-Tests were used to assess statistical differences in measurements between two groups and two-way analysis of variance (ANOVA) were performed when assessing the effects of and interactions between two independent variables. Appropriate post hoc tests were also performed to account for multiple testing. Data are expressed as mean values ± standard error of the mean (SEM). P-values <0.05 were considered statistically significant.

## 3 Results

### 3.1 Modelling CYFIP1-overexpression

**Fig. 1:**
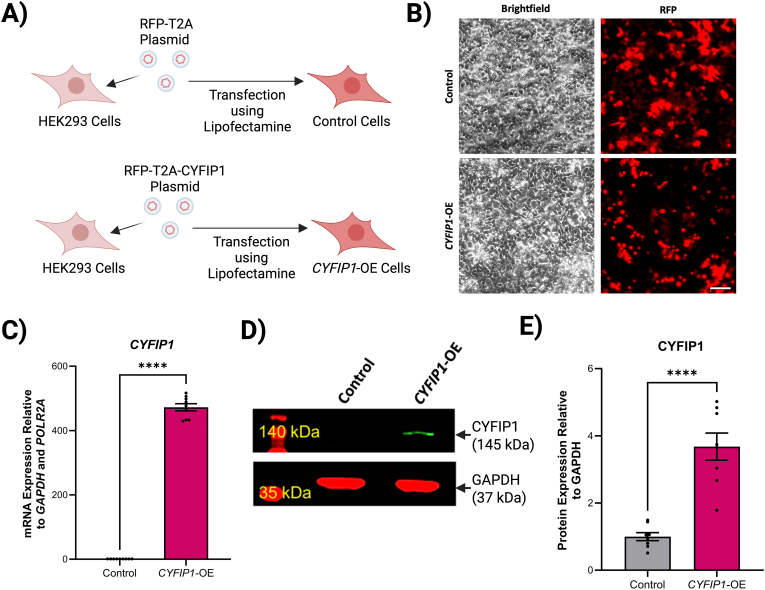
**Modelling CYFIP1 overexpression in HEK-293 cells. A)** HEK-293 cells were transfected with either RFP-T2A plasmid as a control (referred to as Control), or RFP-T2A-CYFIP1 to induce *CYFIP1*-overexpression (OE) (referred to as *CYFIP1*-OE). Created with BioRender.com. **B)** Control and *CYFIP1*-OE cells were imaged 72 h post-transfection live on the Leica DMi8 widefield microscope. Scale bar = 100 μm. **C)** Relative mRNA expression of *CYFIP1* in Control and *CYFIP1*-OE cells 72 h post-transfection measured by qRT-PCR. **D)** Increased CYFIP1 protein expression in *CYFIP1*-OE cells 72 h post-transfection confirmed via Western blotting. **E)** Densitometric analysis of total CYFIP1 protein expression relative to the GAPDH loading control. All values are normalised to the Control cells. For all experiments, values are presented as mean ± SEM of N = 3 independent experiments, 2-3 technical replicates each. Statistical significance against Control was determined using Unpaired Student's T-Tests; ∗∗∗∗P < 0.0001.

To model increased *CYFIP1* expression, we utilised plasmid vectors encoding an RFP-T2A-CYFIP1 construct (to induce *CYFIP1*-OE) or RFP-T2A as a control, which we transfected into HEK-293 cells, referred to as *CYFIP1*-OE and control cells respectively (Fig. 1A and B). We then confirmed increased *CYFIP1* mRNA expression using qRT-PCR (Fig. 1C) and protein expression using Western blotting (Fig. 1D and E). *CYFIP1* mRNA expression was significantly increased in *CYFIP1*-OE cells (mean ± SEM = 472.30 ± 11.20 for *CYFIP1*-OE cells vs 1.00 ± 0.02 for control cells, P < 0.0001), as were CYFIP1 protein levels, compared to control cells (mean ± SEM = 3.68 ± 0.40 for *CYFIP1*-OE cells vs 1.00 ± 0.12 for control cells, P < 0.0001)

### 3.2 *CYFIP1*-OE leads to reduced JAKMIP1 expression

**Fig. 2:**
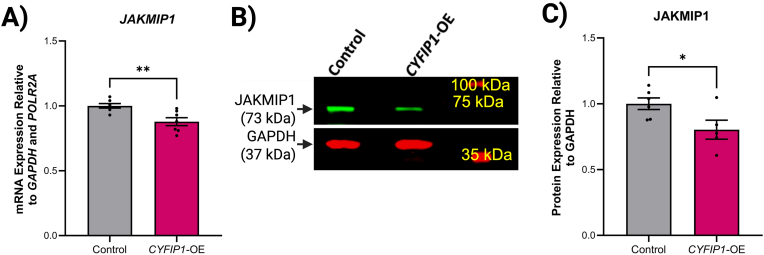
***CYFIP1*-OE leads to reduced JAKMIP1 mRNA and protein expression. A)** Relative mRNA expression of *JAKMIP1* in Control and *CYFIP1*-OE cells measured by qRT-PCR. **B)** Western blotting of JAKMIP1 expression in Control and *CYFIP1*-OE cells. **C)** Densitometric analysis of total JAKMIP1 protein expression relative to the GAPDH loading control. For all experiments, values are presented as mean ± SEM of N = 3 independent experiments, 2-3 technical replicates each. Statistical significance against Control was determined using Unpaired Student's T-Tests; ∗P < 0.05; ∗∗P < 0.01.

It has been previously demonstrated that *JAKMIP1* expression is reduced following *CYFIP1*-OE in human neuroblastoma cells (Nishimura et al., 2007). Therefore, to confirm whether this holds true in HEK-293 cells, we measured *JAKMIP1* expression in *CYFIP1*-OE HEK-293 cells by qRT-PCR and Western blotting. Consistent with Nishimura et al. (2007), we found that *JAKMIP1* mRNA expression was significantly reduced in *CYFIP1*-OE HEK293 cells (Fig. 2A) (mean ± SEM = 0.88 ± 0.03 for *CYFIP1*-OE cells vs 1.00 ± 0.02 for control cells, P = 0.0098), as was JAKMIP1 protein expression (Fig. 2B and C) (mean ± SEM = 0.80 ± 0.07 for *CYFIP1*-OE cells vs 1.00 ± 0.04 for control cells, P = 0.038).

### 3.3 *CYFIP1*-OE alters the expression of several cytokine signaling-related transcription factors

**Fig. 3:**
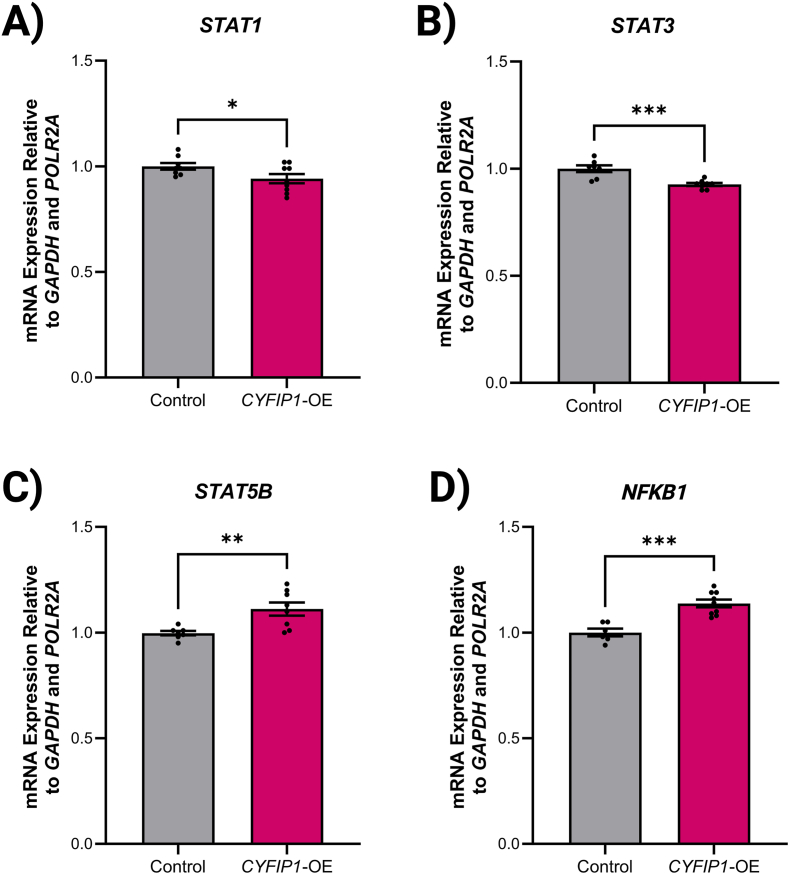
***CYFIP1*-OE alters the expression of other cytokine signaling-related transcription factors.** Relative mRNA expression of **A)***STAT1*, **B)***STAT3*, **C)***STAT5B* or **D)***NFKB1* in Control and *CYFIP1*-OE cells measured by qRT-PCR. For all experiments, values are presented as mean ± SEM of N = 3 independent experiments, 2-3 technical replicates each. Statistical significance against Control was determined using Unpaired Student's T-Tests; ∗P < 0.05; ∗∗P < 0.01; ∗∗∗P < 0.001.

We have previously demonstrated that Dup(15q) iPSCs and iNeurons with increased *CYFIP1* expression show reduced *STAT3* expression (Martin et al., 2025). Furthermore, we have demonstrated that *JAKMIP1*-knockout SH-SY5Y cells also show reduced *STAT3* expression and that *JAKMIP1-*deficiency may alter other cytokine signaling pathways too (Martin et al., 2025). Therefore, to determine whether *CYFIP1*-OE may also lead to altered expression of cytokine signaling-related transcription factors, qRT-PCR was performed to measure mRNA expression of *STAT1*, *STAT3*, *STAT5B* and *NFKB1*. We observed that the expression of *STAT1* (Fig. 3A) and *STAT3* (Fig. 3B) were reduced in *CYFIP1*-OE HEK-293 cells (*STAT1*: mean ± SEM = 0.94 ± 0.02 for *CYFIP1*-OE cells vs 1.00 ± 0.02 for control cells, P = 0.05, *STAT3*: mean ± SEM = 0.93 ± 0.01 for *CYFIP1*-OE cells vs 1.00 ± 0.02 for control cells, P = 0.0007), whereas the expression of *STAT5B* (Fig. 3C) and *NFKB1* (Fig. 3D) were increased (*STAT5B:* mean ± SEM = 1.11 ± 0.031 for *CYFIP1*-OE cells vs 1 ± 0.022 for control cells, P = 0.006, *NFKB1:* mean ± SEM = 1.14 ± 0.02 for *CYFIP1*-OE cells vs 1.00 ± 0.02 for control cells, P = 0.0002). These findings indicate that *CYFIP1*-OE is associated with altered expression of several cytokine signalling-related transcription factors.

### 3.4 *CYFIP1*-OE enhances IL-6-induced STAT3 activity

**Fig. 4:**
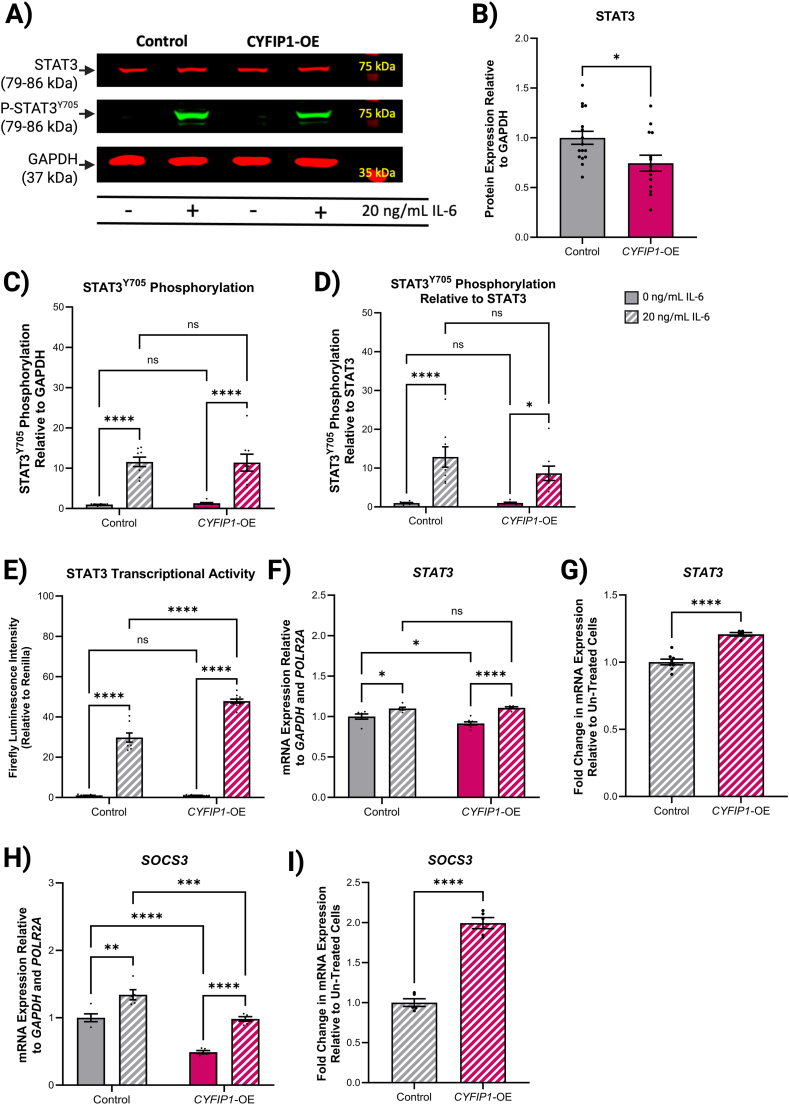
***CYFIP1*-OE enhances STAT3 transcriptional activity but not STAT3 activation. A)** Western blotting of STAT3 expression and phosphorylation (P-STAT3^Y705^) in Control and *CYFIP1*-OE cells following 30-min treatment with 0 ng/mL IL-6 or 20 ng/mL IL-6. Densitometric analysis of **B)** total STAT3 protein expression relative to the GAPDH loading control, **C)** P-STAT3^Y705^ relative to the GAPDH loading control or **D)** P-STAT3^Y705^ relative to total STAT3 protein expression. **E)** Total transcriptional activity at a STAT3-regulated promoter in Control and *CYFIP1*-OE cells stimulated with or without 20 ng/mL IL-6 for 18 h post-transfection with Cignal Reporter plasmids. Firefly luminescence intensity is expressed as a ratio to Renilla luminescence intensity to adjust for transfection efficiency. **F)** Relative mRNA expression of *STAT3* in Control and *CYFIP1*-OE cells treated with 0 ng/mL or 20 ng/mL IL-6 for 4 h, measured by qRT-PCR. **G)** Fold change in *STAT3* mRNA expression following IL-6 treatment relative to untreated cells in part F). Values presented as mean ± SEM of N = 3 independent experiments, 3 technical replicates each. Statistical significance against Control was determined using Unpaired Student's T-Tests; ∗∗∗∗P < 0.0001. **H)** Relative mRNA expression of *SOCS3* in Control and *CYFIP1*-OE cells treated with 0 ng/mL or 20 ng/mL IL-6 for 4 h, measured by qRT-PCR. **I)** Fold change in *SOCS3* mRNA expression following IL-6 treatment relative to untreated cells in part H). For all experiments, values are presented as mean ± SEM of N = 3 independent experiments, 3-4 technical replicates each. Statistical significance against Control was determined using Unpaired Student's T-Tests when comparing two groups, or two-way ANOVAs with Tukey's Honest Significant Difference tests when comparing multiple groups; ns – P > 0.05; ∗P < 0.05; ∗∗P < 0.01; ∗∗∗P < 0.001; ∗∗∗∗P < 0.0001.

To investigate whether reduced *STAT3* expression in *CYFIP1*-OE HEK-293 cells leads to altered cytokine-induced STAT3 responses, we first performed Western blotting to assess IL-6-induced STAT3^Y705^ phosphorylation (representative of STAT3 activation) (Fig. 4A). Despite the reduction in STAT3 expression (Fig. 4B) (mean ± SEM = 0.74 ± 0.08 for *CYFIP1*-OE cells vs 1.00 ± 0.07 for control cells, P = 0.019), *CYFIP1*-OE cells still show strong responses to IL-6, demonstrating no significant difference in IL-6-stimulated STAT3^Y705^ phosphorylation levels compared to control cells, both at the ‘whole-cell level’ (when STAT3^Y705^ phosphorylation levels are normalised to GAPDH) (Fig. 4C) (mean difference = 7.61 for *CYFIP1*-OE cells with vs without IL-6, P = 0.012; mean difference = 11.86 for control cells with vs without IL-6, P < 0.0001), and upon closer examination of the phosphorylation kinetics of STAT3 itself (when STAT3^Y705^ phosphorylation levels are normalised to total STAT3 protein levels) (Fig. 4D) (mean difference = 10.08 for *CYFIP1*-OE cells with vs without IL-6, P < 0.0001; mean difference = 10.60 for control cells with vs without IL-6, P < 0.0001). This suggests that although *CYFIP1*-OE cells show reduced STAT3 expression, STAT3 activation following IL-6 stimulation is unaltered.

STAT3 activity is modulated by various post-translational modifications in addition to phosphorylation at the Y705 residue. Therefore, despite observing no change in STAT3^Y705^ phosphorylation following *CYFIP1*-OE, we examined whether *CYFIP1*-OE cells show altered IL-6-induced transcriptional activity by performing a DLR™ assay. This assay places the control of a Firefly luciferase under a STAT3 response element, meaning that increased Firefly luminescence is indicative of increased STAT3 transcriptional activity. Using this DLR™ assay, we found that *CYFIP1*-OE cells show increased Firefly luminescence following IL-6 stimulation than control cells (Fig. 4E) (mean difference = 46.84 for *CYFIP1*-OE cells with vs without IL-6, P < 0.0001; mean difference = 28.77 for control cells with vs without IL-6, P < 0.0001; mean difference = 18.09 for *CYFIP1*-OE cells with IL-6 vs control cells with IL-6, P < 0.0001), suggesting that IL-6-induced STAT3 transcriptional activity is enhanced in *CYFIP1*-OE cells regardless of the reduced STAT3 expression.

To validate the DLR™ assay results, qRT-PCR was used to measure the mRNA expression of STAT3-responsive genes in *CYFIP1*-OE cells following IL-6 stimulation. Somewhat contradictory to the DLR™ assay, levels of *STAT3* mRNA in IL-6-treated *CYFIP1*-OE cells were not higher than those in IL-6-treated control cells (Fig. 4F) (mean difference = 0.19 for *CYFIP1*-OE cells with vs without IL-6, P < 0.0001; mean difference = 0.10 for control cells with vs without IL-6, P = 0.026; mean difference = 0.01 for *CYFIP1*-OE cells with IL-6 vs control cells with IL-6, P = 0.99). However, considering that *CYFIP1*-OE cells already show reduced *STAT3* mRNA expression at baseline, this is unsurprising. Therefore, after normalising the levels of IL-6-stimulated *STAT3* expression to unstimulated *STAT3* expression, *CYFIP1*-OE cells show an enhancement in IL-6-induced *STAT3* mRNA expression compared to control cells (Fig. 4G) (mean ± SEM = 1.21 ± 0.01 for *CYFIP1*-OE cells vs 1.00 ± 0.02 for control cells, P < 0.0001). The expression of *SOCS3* shows a similar phenomenon whereby IL-6-stimulated *CYFIP1*-OE cells appear to show lower *SOCS3* mRNA levels compared to IL-6-treated control cells (Fig. 4H) (mean difference = 0.49 for *CYFIP1*-OE cells with vs without IL-6, P < 0.0001; mean difference = 0.34 for control cells with vs without IL-6, P = 0.0014; mean difference = 0.36 for *CYFIP1*-OE cells with IL-6 vs control cells with IL-6, P = 0.0009), however the fold change in *SOCS3* expression relative to unstimulated cells shows that IL-6-induced *SOCS3* expression is magnified in *CYFIP1*-OE cells (Fig. 4I) (mean ± SEM = 1.99 ± 0.007 for *CYFIP1*-OE cells vs 1.00 ± 0.05 for control cells, P < 0.0001). Taken together, these findings complement the DLR™ assay results, suggesting that *CYFIP1*-OE cells show enhanced STAT3 transcriptional activity following IL-6 stimulation.

### 3.5 *CYFIP1*-OE alters IL-6-associated neurite outgrowth in SH-SY5Y cells

**Fig. 5:**
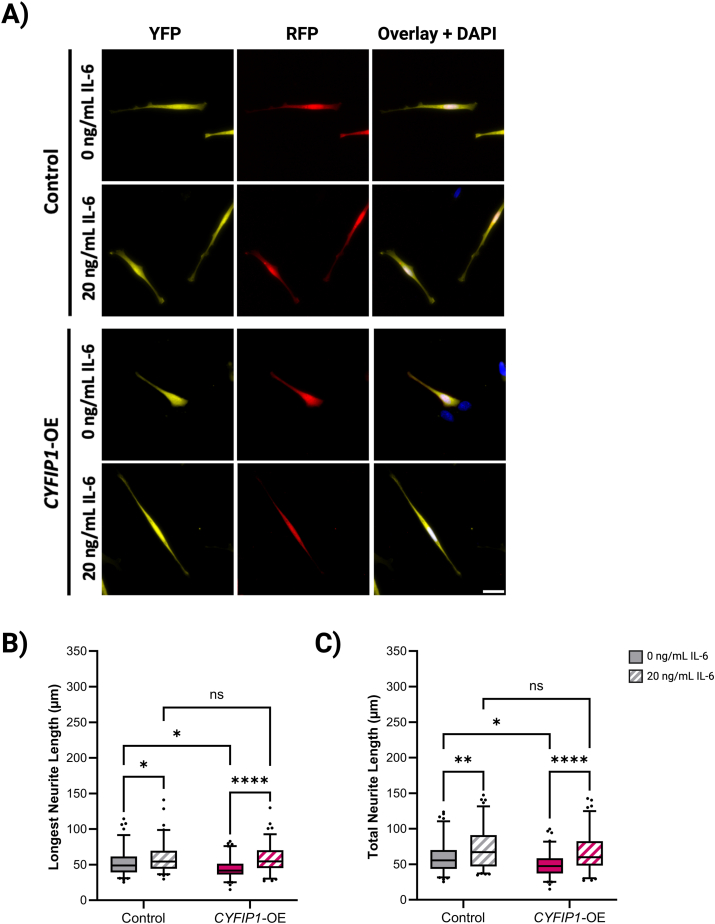
*CYFIP1*-OE enhances IL-6-induced neuritogenesis. SH-SY5Y cells were Nucleofected with either the RFP-T2A backbone vector and pCAβ-YFP as the control condition; or RFP-T2A-CYFIP1 and pCAβ-YFP to assess the effects of CYFIP1-OE on IL-6-influenced neuritogenesis. Cells were differentiated for two days with 20ng/mL BDNF and 10μM RA under serum starvation with or without 20ng/mL IL-6, fixed with 4% PFA and stained with DAPI. **A)** Fluorescence micrographs of differentiated SH-SY5Y cells. Images captured using an upright Leica DM4000 B LED microscope. Scale bar = 50μm. Neurite tracing analysis was performed using the FIJI software. **B)** The length of the longest neurite produced by the cell was measured for all conditions. **C)** The sum length of all neurites produced per cell is presented for each condition. For all experiments, values are presented as mean ± SEM of N = 4 independent experiments, 3 technical replicates each. Statistical significance against Control was determined using two-way ANOVAs with Tukey's Honest Significant Difference tests; ns – P > 0.05; ∗P < 0.05; ∗∗P < 0.01; ∗∗∗∗P < 0.0001.

To determine whether *CYFIP1*-OE is associated with altered IL-6-related neurite outgrowth, we differentiated control and *CYFIP1*-OE SH-SY5Y cells in the absence or presence of IL-6 and measured longest neurite length (LNL) and total neurite length (TNL) (Fig. 5A), as we have previously shown that IL-6 induces neurite outgrowth in SH-SY5Y cells (Martin et al., 2025). As expected, control and *CYFIP1*-OE cells differentiated in the presence of IL-6 exhibit longer LNL (Fig. 5B) and TNL (Fig. 5C) measurements than cells differentiated without IL-6. *CYFIP1*-OE cells appear to have enhanced neurite outgrowth responses to IL-6, as although *CYFIP1*-OE cells produce shorter neurites compared to the control as baseline, the LNL (Fig. 5B) and TNL (Fig. 5C) measurements are no longer significantly different between the two populations when differentiated with IL-6 (LNL: mean difference = 13.60 for *CYFIP1*-OE cells with vs without IL-6, P < 0.0001; mean difference = 7.47 for control cells with vs without IL-6, P = 0.025; mean difference = 7.12 for *CYFIP1*-OE cells without IL-6 vs control cells without IL-6, P = 0.039; mean difference = 0.99 for *CYFIP1*-OE cells with IL-6 vs control cells with IL-6, P = 0.98) (TNL: mean difference = 17.38 for *CYFIP1*-OE cells with vs without IL-6, P < 0.0001; mean difference = 12.47 for control cells with vs without IL-6, P = 0.0024; mean difference = −10.00 for *CYFIP1*-OE cells without IL-6 vs control cells without IL-6, P = 0.026; mean difference = -5.08 for *CYFIP1*-OE cells with IL-6 vs control cells with IL-6, P = 0.47). These data indicate that *CYFIP1*-OE is associated with reduced basal neurite outgrowth in SH-SY5Y cells, and while IL-6 treatment increases neurite outgrowth in both control and *CYFIP1*-OE cells, IL-6 treatment reduces the baseline difference between the two groups, suggesting that *CYFIP1*-OE may be enhancing IL-6-associated neurite outgrowth.

### 3.6 *CYFIP1*-OE enhances IFN-γ-induced STAT1 transcriptional activity

**Fig. 6:**
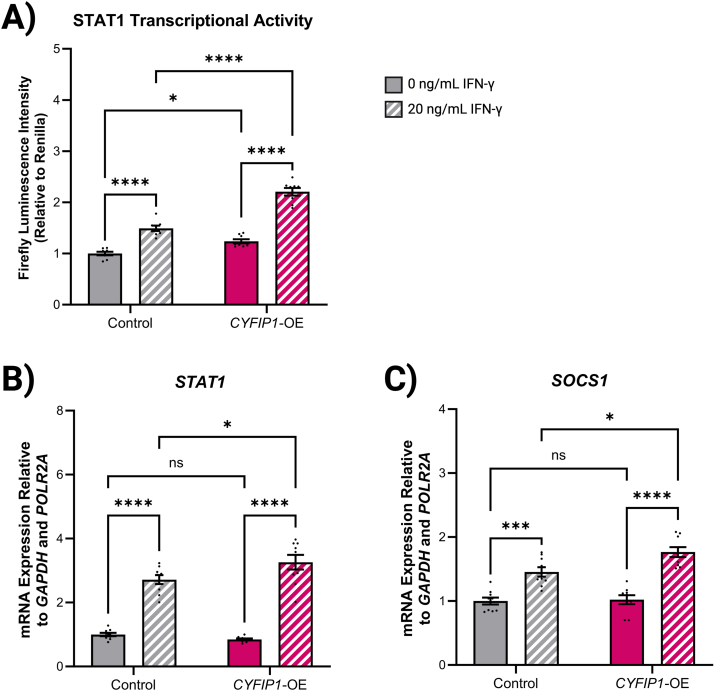
*CYFIP1*-OE leads to enhanced STAT1 transcriptional activity following IFN-γ treatment. **A)** Total transcriptional activity at a STAT1-regulated promoter in Control and *CYFIP1*-OE cells stimulated with or without 20ng/mL IFN-γ for 18 h post-transfection with Cignal Reporter plasmids. Firefly luminescence intensity is expressed as a ratio to Renilla luminescence intensity to adjust for transfection efficiency. **B)** Relative mRNA expression of *STAT1* in Control and *CYFIP1*-OE cells treated with 0ng/mL or 20ng/mL IFN-γ for 4 h, measured by qRT-PCR. **C)** Relative mRNA expression of SOCS1 in Control and CYFIP1-OE cells treated with 0ng/mL or 20ng/mL IFN-γ for 4 h, measured by qRT-PCR. For all experiments, values are presented as mean ± SEM of N = 4 independent experiments, 3 technical replicates each. Statistical significance against Control was determined using two-way ANOVAs with Tukey's Honest Significant Difference tests; ns – P > 0.05; ∗P < 0.05; ∗∗∗P < 0.001; ∗∗∗∗P < 0.0001.

Having determined that *CYFIP1*-OE alters the expression of several cytokine signaling-related transcription factors, and that *CYFIP1*-OE enhances IL-6-induced STAT3 activity, we aimed to investigate whether *CYFIP1*-OE alters the activity of another cytokine signaling-related transcription factor. STAT1 was chosen to follow up with because similar to *STAT3* expression, the expression of *STAT1* was also reduced in *CYFIP1*-OE HEK-293 cells. A DLR™ assay which placed the control of a Firefly luciferase under a GAS element (the response element STAT1 binds to initiate transcription) was performed. We found that *CYFIP1*-OE HEK-293 cells show increased Firefly luminescence following IFN-γ stimulation compared to control cells (Fig. 6A) (mean difference = 0.97 for *CYFIP1*-OE cells with vs without IFN-γ, P < 0.0001; mean difference = 0.49 for control cells with vs without IFN-γ, P < 0.0001; mean difference = 0.72 for *CYFIP1*-OE cells with IFN-γ vs control cells with IFN-γ, P < 0.0001), suggesting that IFN-γ-induced STAT1 transcriptional activity is enhanced in *CYFIP1*-OE cells, again regardless of the reduced *STAT1* expression.

As validation of the DLR™ assay, qRT-PCR was performed to measure the expression of STAT1-responsive genes following IFN- γ stimulation. *CYFIP1*-OE cells show increased IFN-γ-induced *STAT1* expression (Fig. 6B) (mean difference = 2.41 for *CYFIP1*-OE cells with vs without IFN-γ, P < 0.0001; mean difference = 1.71 for control cells with vs without IFN-γ, P < 0.0001; mean difference = 0.54 for *CYFIP1*-OE cells with IFN-γ vs control cells with IFN-γ, P = 0.049), suggesting an enhanced transcription response to IFN-γ stimulation. Additionally, although not significantly different at baseline, *CYFIP1*-OE cells show increased *SOCS1* expression following IFN-γ treatment (Fig. 6C) (mean difference = 0.74 for *CYFIP1*-OE cells with vs without IFN-γ, P < 0.0001; mean difference = 0.46 for control cells with vs without IFN-γ, P = 0.0003; mean difference = 0.31 for *CYFIP1*-OE cells with IFN-γ vs control cells with IFN-γ, P = 0.018), again suggesting an enhanced STAT1 transcriptional response to IFN-γ stimulation. This also suggests that the observed alterations in IL-6/STAT3 signaling are not limited to STAT3 and that CYFIP1 may play a role in modulating a larger range of cytokine signaling pathways.

## 4 Discussion

### 4.1 A novel role for CYFIP1 in modulating IL-6/STAT3 signaling independent from JAKMIP1

Nishimura et al. (2007) first linked *JAKMIP1* to ASD and Dup(15q), reporting that *JAKMIP1* expression was altered in Dup(15q) and that overexpression of the chromosome 15q gene *CYFIP1* correspondingly resulted in *JAKMIP1* downregulation. We expanded upon this in our previous work, identifying JAKMIP1 as a modulator of IL-6 cytokine signaling in SH-SY5Y cells, and demonstrating that Dup(15q) iPSCs and cortical neurons with elevated *CYFIP1* dosage and expression exhibit enhanced IL-6-induced STAT3 activity (Martin et al., 2025). However, given that the magnified IL-6 signaling observed in these Dup(15q) cells did not correlate with *JAKMIP1* expression levels, we hypothesised that another gene within the 15q region, likely *CYFIP1*, might be driving this increased responsiveness to IL-6.

To test this hypothesis, we induced *CYFIP1-*overexpression in HEK-293 cells and discovered that this resulted in the altered expression of several cytokine signaling-related transcription factors. In particular, *STAT1* and *STAT3* expression were reduced, whereas *STAT5B* and *NFKB1* expression were raised, suggesting that CYFIP1 may potentially exert broad cellular functions that impact the signaling networks of multiple cytokines. Taking this forward, we measured IL-6-induced activation and activity of STAT3. Consistent with the Dup(15q) iPSCs and neurons from our previous work, we observed that despite the reduced *STAT3* expression, IL-6-induced STAT3 responses were enhanced in *CYFIP1*-OE HEK-293 cells. Furthermore, *CYFIP1-*overexpression also potentiated IL-6-triggered neuritogenesis in SH-SY5Y cells. As such, this study provides the first evidence that CYFIP1 may play a role in regulating IL-6/STAT3 signaling in both HEK-293 and SH-SY5Y cells, and its upregulation could be responsible for the enhanced responsiveness to IL-6 exhibited by Dup(15q) iPSCs and neurons.

### 4.2 How does CYFIP1 influence STAT1 and STAT3 activity?

The mechanisms underlying CYFIP1-mediated regulation of *STAT1* and *STAT3* expression and activity remain unclear. Regarding STAT3, reduced *STAT3* expression in *JAKMIP1*-deficient cells (Martin et al., 2025) may suggest that JAKMIP1, rather than CYFIP1, is responsible for regulating *STAT3* expression. This could occur through its interactions with various RNA-binding proteins or splicing factors (Martin et al., 2025). However, it is important to note that *CYFIP1-*overexpression enhances IL-6-induced STAT3 transcriptional activity, whereas *JAKMIP1*-knockout impairs it (Martin et al., 2025). Thus, whilst JAKMIP1 could be responsible for controlling *STAT3* expression, when considering STAT3 activity, our results indicate that it is more likely that CYFIP1 is responsible by a mechanism independent of JAKMIP1.

Interestingly, despite *CYFIP1-*overexpression leading to increased STAT3 transcriptional activity following IL-6 treatment, we observed no significant difference in the levels STAT3^Y705^ phosphorylation in *CYFIP1*-OE cells, suggesting that CYFIP1 does not alter STAT3 phosphorylation kinetics, and thus STAT3 activation. One reason for this discrepancy could be because of the influence of other post-translational modifications of STAT3. For example, STAT3^K685^ acetylation may be necessary for IL-6-induced STAT3-responsive gene expression (Wang et al., 2005). Rather than modulating STAT3 activation through phosphorylation, perhaps CYFIP1 may somehow control STAT3 activation through acetylation, however, the exact mechanism by which CYFIP1 could regulate acetylation is unclear.

In terms of *STAT1* expression and activity, it would not be unreasonable to suggest that these changes in STAT1 activity may be mediated by the changes in *JAKMIP1* expression. JAKMIP1 was previously reported to inhibit Interferon-α (IFN-α) signaling (Steindler et al., 2004b). Importantly, both IFN-α and IFN-γ signaling cascades involve the phosphorylation and activation of STAT1. It is therefore likely that CYFIP1-induced downregulation of *JAKMIP1* may reduce the inhibiting influence of JAKMIP1 on IFN signaling, thereby enhancing the IFN response. Furthermore, STAT1 activity is also known to be altered by acetylation, similar to STAT3 (Leslie et al., 2024). If CYFIP1-mediated regulation of STAT3 is dependent on post-translational modifications, the same mechanism(s) could be responsible for CYFIP1 regulation of both STAT3 and STAT1 activity. Of course, all of these possibilities would require further validation.

### 4.3 Relevance to ASD and neurodevelopment

Cytokine signaling plays crucial roles in neurodevelopment beyond immune responses, with STAT3 and STAT1 signaling in particular regulating several key processes. For example, STAT3 signaling has been demonstrated to control the switch between neurogenesis and gliogenesis, promoting both neural progenitor cell self-renewal and astrocyte differentiation (Hong and Song, 2014). Similarly, STAT1 signaling has been shown to be involved in neurogenesis, and promoting neurite outgrowth (Warre-Cornish et al., 2020). Given how crucial STAT3 and STAT1 signaling are for neurodevelopment, it is unsurprising that alterations in STAT3 and STAT1 signaling are associated with Neurodevelopmental disorders such as ASD (Garay and McAllister, 2010; Majerczyk et al., 2022). MIA, where an immune response is triggered by infection or exposure to immunogenic material during pregnancy, is associated with increased incidence of ASD (Choudhury and Lennox, 2021). Both IL-6 and IFN-γ are pro-inflammatory cytokines that have been implicated in the pathogenesis of ASD, with IL-6 being a key player in MIA-induced ASD-like behaviours (Choudhury and Lennox, 2021; Samuelsson et al., 2006). Considering that *CYFIP1*-OE enhances STAT3 and STAT1 responses to IL-6 and IFN-γ respectively suggests that CYFIP1 may play a pivotal role in amplifying the inflammatory signals during MIA, potentially exacerbating the effects of immune activation on foetal brain development.

Amplification of both the IL-6/STAT3 and IFN-γ/STAT1 signaling pathways by CYFIP1 provides a potential molecular mechanism that explains the overlap between immune activation and ASD, particularly in cases where no obvious inflammatory trigger is identified. Elevated cytokine levels are frequently reported in individuals with ASD (Ashwood et al., 2011), and our results suggest that *CYFIP1-*overexpression in certain cases of syndromic ASD could act as a molecular convergence point, increasing the sensitivity of the developing brain to immune signals even under baseline inflammatory conditions. This could help to explain cases of ASD where immune activation is not readily apparent but inflammation-induced neurodevelopmental disruption still occurs.

### 4.4 Limitations to this study

HEK-293 cells were used in this study as they are easy to transfect and thus well suited to enable transient *CYFIP1-*overexpression (Thomas and Smart, 2005). As we are examining the role of CYFIP1 in the context of ASD and neural cytokine signaling, HEK-293 cells may not have been the most representative model cell line due to their renal origin. However, despite being an embryonic kidney cell line, there is some evidence to suggest that HEK-293 cells display neural characteristics, expressing many neuronal genes, including those encoding neurofilament proteins and neuronal receptors (He and Soderlund, 2010; Shaw et al., 2002). Therefore, although HEK-293 cells are non-neuronal, they can be used for their neuronal progenitor cell-like properties (He and Soderlund, 2010; Shaw et al., 2002). Importantly, we also tested the potential effect of altered IL-6/STAT3 signaling on a neuronal behaviour using differentiated SH-SY5Y cells, therefore validating our findings in a more neuronal-relevant model. It was also beneficial to use SH-SY5Y cells so that we could directly compare the effects of *CYFIP1*-OE on IL-6-stimulated neurite outgrowth to *JAKMIP1* deficiency as per our previous work (Martin et al., 2025). Yet, it is also important to acknowledge that SH-SY5Y cells are not a perfect model (Kovalevich and Langford, 2013) and it is imperative that these findings are replicated in a more mature neuronal model to test direct relevancy for ASD.

In addition to choice of cell lines, there are also limitations to consider with the methodologies implemented to study cytokine signaling pathways. First is the choice of cytokine concentration. Cells were treated with 20 ng/mL IL-6 or IFN-γ, as we have previously found these concentrations to be sufficient to induce a robust STAT3 or STAT1 activation (Martin et al., 2025; Dhayal et al., 2022), yet these concentrations are higher even than the serum concentrations of IL-6 or IFN-γ observed in individuals with sepsis (Hou et al., 2015; Gharamti et al., 2022). Although supraphysiological, these concentrations ensure a strong signalling response, facilitating the detection of conditions that may modify these signalling pathways. Another important consideration when investigating cytokine signaling pathways is the choice of timepoints for cytokine treatments. All cytokine treatment timepoints were also chosen based on previous work (Martin et al., 2025; Dhayal et al., 2022). However, it is important to note that STAT responses to cytokine stimulation fluctuate, and that they tend to be self-propagating responses (Braun et al., 2013; Garbers et al., 2015). Hence, it is possible that varying cytokine concentrations or the duration of treatments may reveal additional insights into how these signaling pathways are altered in cases of ASD involving alterations to *CYFIP1* expression and function (or its related pathways).

## 5 Conclusion

In summary, this study identifies a novel role for CYFIP1 in regulating the expression of cytokine signaling-related transcription factors, as well as enhancing their activity following cytokine stimulation. By amplifying these inflammatory pathways, CYFIP1 could potentially enhance neuroinflammatory signaling, altering neurodevelopment and increasing susceptibility in certain syndromic forms of ASD. Further research is required to understand the mechanism by which CYFIP1 modulates these cytokine signaling pathways and to investigate this in the context of ASD with MIA.

## Ethics approval and consent to participate

Not applicable.

## Consent for publication

Not applicable.

## Availability of data and materials

The datasets used and/or analysed during the current study are available from the corresponding author on reasonable request.

## Funding

This work was supported by a Wellcome Wellcome Trust funded Translational Research Exchange @ Exeter (TREE) pump-priming fund and the Bristol Japanese Cultural Showcase.

## CRediT authorship contribution statement

**Emily-Rose Martin:** Conceptualization, Investigation, Methodology, Validation, Visualization, Writing – original draft, Writing – review & editing. **Josan G. Martin:** Conceptualization, Methodology, Writing – review & editing. **Mark A. Russell:** Supervision, Writing – review & editing. **Asami Oguro-Ando:** Conceptualization, Funding acquisition, Supervision, Writing – review & editing.

## Declaration of competing interest

The authors declare that they have no known competing financial interests or personal relationships that could have appeared to influence the work reported in this paper.

## Data Availability

Data will be made available on request.

## List of abbreviations

ASD: Autism spectrum disorder
Dup(15q): Chromosome 15q-duplication syndrome
CYFIP1: Cytoplasmic FMR1-intercating protein 1
iPSC: Induced pluripotent stem cell
IL-6: Interleukin-6
STAT3: Signal transducer and activator of transcription 3
OE: Overexpression
MIA: Maternal immune activation
JAKMIP1: Janus kinase and microtubule-interacting protein 1
FXS: Fragile X syndrome
FMRP: Fragile X messenger ribonuclear protein
WRC: WAVE regulatory complex
SMAD3: SMAD family member 3
NF-kB: Nuclear factor of κB
TGF-β1: Transforming growth factor β1
GAPDH: Glyceraldehyde-3-phosphate dehydrogenase
DLR™: Dual-luciferase reporter™
SOCS3: Suppressor of cytokine signaling 3
LNL: Longest neurite length
TNL: Total neurite length
IFN-γ: Interferon-γ
RA: Retinoic acid
BDNF: Brain-derived neurotrophic factor
HEK: Human embryonic kidney
FBS: Foetal bovine serum
JAK: Janus Kinase
RFP: Red fluorescent protein
YFP: Yellow fluorescent protein
GAS: Gamma-interferon-activated site
PDL: Poly-d-lysine
DAPI: 4',6-diamidine-2-phenylindone
POLR2A: RNA polymerase II subunit A
