# Supplementary material for: CYFIP1 overexpression amplifies IL-6/STAT3 and IFN-γ/STAT1 signaling: potential implications for neuroinflammation and autism spectrum disorder
Source: Brain Behav Immun Health. 2026 Jun 22;55:101293. doi: 10.1016/j.bbih.2026.101293 (PMC13316639; doi:10.1016/j.bbih.2026.101293)
Supplement: Multimedia component 1 [file mmc1.docx]

**Supplementary File 1: Supplementary Methods**

**HEK-293 and SH-SY5Y cell culture**

Human embryonic kidney (HEK)-293 cells (#CRL-1573™, *American Type Culture Collection*) and human neuroblastoma cells (#CRL-2266™, *American Type Culture Collection*) were thawed from frozen stocks stored in 10% dimethyl sulfoxide (#D2650-5X5ML, *Sigma-Aldrich*®) at −80°C. HEK-293 cells and SH-SY5Y cells were maintained in 10-cm diameter Petri dishes (#P7612-360EA, *Sigma-Aldrich*®) with 10 mL Dulbecco’s modified eagle medium/nutrient mixture F-12 with GlutaMAX (referred to henceforth as DMEM/F-12) (#11524436, *Fisher Scientific*™) supplemented with 10% foetal bovine serum (FBS) (#11550356, *Fisher Scientific*™), and incubated at 37°C, 5% CO_2_, 95% humidity. Cells were passaged at 70-80% confluency using 3 mL of pre-warmed TrypLE™ (#10718463, *Fisher Scientific*™) for 5 min and TrypLE™ was inactivated by dilution in an equal volume of DMEM/F-12 + 10% FBS.

**Cytokine Treatments**

Cells were stimulated with 20 ng/mL IL-6 (#7270IL-025, *R&D Systems*) or 20 ng/mL Interferon-γ (IFN-γ) (#285-IF-100, *R&D Systems*) diluted in DMEM/F-12 + FBS (or in DMEM/F-12 supplemented with Retinoic Acid (RA) and Brain-Derived Neurotrophic Factor (BDNF) for neurite tracing experiments). These cytokine concentrations and time-points were chosen as we have previously found them to be sufficient for inducing a STAT3 or STAT1 response (17,24). For all experiments using IL-6 or IFN-γ, control cells were used which were treated with corresponding media not containing IL-6 or IFN-γ. Cells were treated with IL-6 or IFN-γ for different amounts of time depending on the purpose of the experiment. To assess STAT3 activation by Western blotting for phosphorylated STAT3 (P-STAT3^Y705^), cells were treated with IL-6 for 30 minutes. To assess STAT3 or STAT1 transcriptional activity by Dual-Luciferase® Reporter (DLR™) assays, cells were treated with IL-6 or IFN-γ for 18 hours post-transfection. To measure mRNA expression of STAT3 or STAT1 responsive genes, cells were treated with IL-6 or IFN-γ for 4 hours. Finally, to assess IL-6-induced neurite outgrowth, cells were treated with IL-6 over a 48-hour period of differentiation. A schematic overview of the cytokine treatment conditions and time-points can be found in **Supplementary Figure 1**.

**HEK-293 cell transfection**

24 hours prior to transfection, 300,000 HEK-293 cells were seeded into Nunc cell-culture treated 6-well plates (#10469282, *Fisher Scientific*™) in 3 mL DMEM/F-12 + 10% FBS. The following day, HEK-293 cells were transfected with either an RFP-T2A-CYFIP1 plasmid to induce *CYFIP1*-overexpression (these cells are referred to as ‘*CYFIP1*-OE cells), or the backbone RFP-T2A plasmid as a control (these cells are referred to as ‘control cells’) using Lipofectamine™ LTX Reagent (#15338100, *Fisher Scientific*™) following manufacturer instructions (i.e., final amount of 2.5 μg of plasmid and 5 μL of Lipofectamine™ LTX per well). Media was replaced the following day. All experiments were performed 72 hours post-transfection.

**SH-SY5Y cell transfection**

2,000,000 SH-SY5Y cells were transfected by Nucleofection™ (#V4XC-2012, *Lonza Bioscience*) following manufacturer instructions with 5 μg of either a mixture of the RFP-T2A backbone vector and pCAβ-YFP (encoding yellow fluorescent protein (YFP)) in a 1:1 ratio as the control condition; or a mixture of RFP-T2A-CYFIP1 and pCAβ-YFP (4:1 ratio) to assess the effects of *CYFIP1*-OE on neuritogenesis. A pCAβ-YFP plasmid was co-transfected to enable better visualisation of the cells for neurite tracing as the RFP encoded by the *CYFIP1*-OE plasmid photo-bleaches quickly. Media was replaced the following day.

**Protein extraction and Western blotting**

72 hours post-transfection, cells were washed with ice-cold 1x phosphate buffered saline (1xPBS) (#P4417-50TAB, Sigma-Aldrich®) then collected with a cell scraper in 300 μL of ice-cold RIPA lysis buffer (150 mM NaCl, 50 mM Tris/Cl pH = 8.0, 0.5% Sodium Deoxycholate, 0.1% SDS, 1% Triton X-100) supplemented with 1 mM PMSF, 1% Phosphatase Inhibitor Cocktail 2 (#P5726-1ML, Sigma Aldrich®), 1% Phosphatase Inhibitor Cocktail 3 (#P0044-1ML, Sigma Aldrich®) and 1% Complete Protease Inhibitor Cocktail (#P8340-1ML, Sigma Aldrich®). Cell lysates were incubated on ice for 30 minutes, cleared by centrifugation at 17,000xg for 15 minutes at 4°C, then supernatants collected. Bicinchoninic Acid assays (#10678484, *Thermo Scientific*) were performed to estimate protein concentrations and 50 μg of total protein was diluted with Laemmli buffer (final concentration of 60 mM Tris/Cl pH = 6.8, 10% Glycerol, 2% SDS, 5% 2-Mercaptoethanol, 0.02% Bromophenol Blue), then boiled for 5 minutes at 95°C. 50 μg of total protein for each sample was loaded into 4–15% precast polyacrylamide gels (#4561085, *Bio-Rad Laboratories*) alongside a pre-stained protein ladder (#PL00001, *Proteintech Group*). Electrophoresis was performed in the Mini-PROTEAN® Tetra Vertical Electrophoresis Cell (#1658004, *Bio-Rad Laboratories*) with 1×SDS-PAGE running buffer (2.5 mM tris-base; 19 mM glycine (#10070150, *Fisher Chemical*™); 0.01% SDS; pH = 8.3 in ddH_2_O). Proteins were then transferred onto methanol-activated PVDF membranes (#IPVH304F0, *Millipore*) by a wet transfer method in the Mini-PROTEAN® Tetra Vertical Electrophoresis Cell. PVDF membranes were then blocked for at least 1 hour at room temperature with 5 mL of 5% skim milk in 1x tris-buffered saline (TBS; 2 mM Tris-Base; 15 mM NaCl; pH = 7.6 in ddH_2_O) supplemented with 0.1% Tween® 20 (#10485733, *Fisher* *BioReagents*™) (1xTBS-T). After blocking, membranes were sequentially incubated with 5 mL of primary antibody (gentle rocking for 2-3 hours at room temperature or overnight at 4°C), then secondary antibody (gentle rocking for 30 minutes-1 hour at room temperature). Primary and secondary antibodies were diluted in 5% skim milk in 1x TBS-T (or 5% BSA in 1x TBS-T for anti-P-STAT3^Y705^). The dilution factors and specific antibodies used are listed in **Table 1**. Three 5-minute washes with 1x TBS-T were performed between antibody incubations. Glyceraldehyde-3-Phosphate Dehydrogenase (GAPDH) was used as a loading control to normalise expression of target proteins. Proteins were visualised using the Odyssey® CLx Imager (*LI-COR Biosciences*) and the Image Studio software (Version 5.2; *LI-COR Biosciences*) was used to perform densitometric analyses to compare the abundance of target proteins between samples. Full uncropped Western blot images for each primary antibody used can be found in **Supplementary Figures 1-3**.

| **Catalogue Number** | **Supplier** | **Host Species** | **Target** | **Dilution** | **Conjugated Fluorophore** |
| --- | --- | --- | --- | --- | --- |
| ab156016 | *Abcam* | Rabbit | CYFIP1 | 1:1000 |  |
| sc-47724 | *Santa Cruz Biotechnology* | Mouse | GAPDH | 1:1000 |  |
| 13846-1-AP | *Proteintech* | Rabbit | JAKMIP1 | 1:1000 |  |
| 9139S | *Cell Signaling Technology* | Mouse | STAT3 | 1:1000 |  |
| 9131l | *Cell Signaling Technology* | Rabbit | P-STAT3^Y705^ | 1:1000 |  |
| 35519 | *Invitrogen* | Goat | Mouse | 1:5000 | DyLight™ 680 |
| SA5-10036 | *Invitrogen* | Goat | Rabbit | 1:5000 | DyLight™ 800 |

***Table 1: Antibodies used in this study.***

**RNA extraction, cDNA conversion and qRT-PCR**

72 hours post-transfection, total RNA was isolated and purified using the Direct-zol™ RNA Miniprep kit following manufacturer instructions (#R2051, *Zymo Research*). 500 ng of RNA (purity and concentration determined with a NanoDrop™ ND-8000 spectrophotometer (*Thermo Fisher Scientific™*)) was converted to cDNA using PrimeScript™ RT reagent kit (#RR037A, *Takara Bio Europe*) following manufacturer instructions. qRT-PCR was then performed using HOT FIREPol® EvaGreen® qPCR Master Mix with ROX (#01-02-00500, *Solis BioDyne*) with the QuantStudio 12K Flex qPCR machine (*Thermo Fisher Scientific*™). For primer sequences, see **Table 2**. Each reaction was run in triplicate, and mRNA expression levels were normalised against the geometric mean of two reference genes, *GAPDH* and *RNA Polymerase II Subunit A* (*POLR2A*) using the Pfaffl method (25). Gene expression ratios were then presented in figures relative to the control cells.

| **Gene** | **Strand** | **Sequence** |
| --- | --- | --- |
| *CYFIP1* | Forward | TAC GAG ACG CTG CTG AAG CAG A |
|  | Reverse | TCG TCC AAT CGC CAG TTC TAG G |
| *GAPDH* | Forward | TCC TCT GAC TTC AAC AGC GAC |
|  | Reverse | GCT GTA GCC AAA TTC GTT GTC A |
| *NF-KB* | Forward | GCA GCA CTA CTT CTT GAC CAC C |
|  | Reverse | TCT GCT CCT GAG CAT TGA CGT C |
| *POLR2A* | Forward | CCA TCA AGA GAG TCC AGT TCG |
|  | Reverse | ACC CTC CGT CAC AGA CAT TC |
| *SOCS1* | Forward | TTC GCC CTT AGC GTG AAG ATG G |
|  | Reverse | TAG TGC TCC AGC AGC TCG AAG A |
| *SOCS3* | Forward | CCA AGG ACG GAG ACT TCG ATT C |
|  | Reverse | GGG AAA CTT GCT GTG GGT GA |
| *STAT1* | Forward | ATG GCA GTC TGG CGG CTG AAT T |
|  | Reverse | CCA AAC CAG GCT GGC ACA ATT G |
| *STAT3* | Forward | GGG AAG AAT CAC GCC TTC TAC |
|  | Reverse | ATC TGC TGCT TCT CCG TCA C |
| *STAT5B* | Forward | GCC ACT GTT CTC TGG GAC AAT G |
|  | Reverse | ACA CGA GGT TCT CCT TGG TCA G |

***Table 2: Primers used in this study.***

**Dual-luciferase reporter assays**

50,000 HEK-293 cells were seeded into 24-well plates and incubated overnight. The following day, cells were transfected with 2 μL of STAT3 Cignal Reporter plasmid (#336841, GeneGlobe ID: CCS-9028L, *Qiagen*) or 2 μL of GAS Cignal Reporter plasmid (#336841, GeneGlobe ID: CCS-009L, *Qiagen*) using Lipofectamine™ LTX as previously described. Four hours post-transfection, cells were treated with DMEM/F-12 +10% FBS with or without IL-6- or IFN-γ and incubated overnight. 18 hours later, Firefly and Renilla luciferase activities were measured using the Dual-Luciferase® Reporter (DLR™) Assay System (#E1960, *Promega*) following manufacturer instructions. Luminescence was measured in white-bottomed LUMITRAC™ 96-well plates (#655074, *Greiner Bio-One*) using the PHERAstar FS microplate reader (*BMG LABTECH*).

**SH-SY5Y differentiation and neurite outgrowth analysis**

Sterile 13-mm-diameter glass coverslips (#631-1578, *VWR*®) were placed into 24-well plates and coated with 300 μL of 20 μg/mL poly-D-Lysine (PDL; #354210, *Corning*®) by overnight incubation at 37°C. 24 hours post-transfection, 30,000 SH-SY5Y cells were seeded onto PDL-coated coverslips in DMEM/F-12 + 10% FBS. The following day, media was replaced with 20 ng/mL BDNF and 10 μM RA in DMEM/F-12 (without FBS). 48 hours later, SH-SY5Y cells were fixed with 4% paraformaldehyde (PFA), and nuclei were counterstained with 4′,6-diamidino-2-phenylindole (DAPI) (#D9542, *Sigma-Aldrich*®) for 15 minutes. Coverslips were mounted onto glass microscope slides with ProLong™ Diamond Antifade Mountant (#15468070, *Fisher Scientific*™) and slides were imaged on the inverted DMi8 widefield microscope (*Leica Microsystems*).

All image analysis was performed manually using the *FIJI* software (*ImageJ*; Version 2.9.0; (26)). Prior to image analysis, a scale bar was used to set a pixel to μm ratio to each image, after which the “Segmented Line” tool was used to measure the distance from the edge of the nucleus to the furthest end of each neurite. The length of the longest neurite produced by a single cell (referred to as longest neurite length (LNL); a measure of neurite extension) and the sum length of all neurites produced by a single cell (referred to as total neurite length (TNL); a measure of neuritogenesis) were measured.

**Statistical analysis**

Statistical analyses were performed using GraphPad Prism (GraphPad Prism Version 9.0.0 for Windows, GraphPad Software, San Diego, California USA, www.graphpad.com). Unpaired Student’s t-Tests were used to assess statistical differences in measurements between two groups (e.g., *CYFIP1*-OE against control). When investigating the effects of and interactions between two independent variables (e.g., the effect of both *CYFIP1*-OE and IL-6 treatment on STAT3 transcriptional activity), two-way analysis of variance (ANOVA) were performed. Appropriate post hoc tests were also performed to account for multiple testing. Data are expressed as mean values ± standard error of the mean (SEM). P-values < 0.05 were considered statistically significant.
