# Supplementary material for: CYFIP1 overexpression amplifies IL-6/STAT3 and IFN-γ/STAT1 signaling: potential implications for neuroinflammation and autism spectrum disorder
Source: Brain Behav Immun Health. 2026 Jun 22;55:101293. doi: 10.1016/j.bbih.2026.101293 (PMC13316639; doi:10.1016/j.bbih.2026.101293)
Supplement: Multimedia component 2 [file mmc2.docx]

**Supplementary Files**


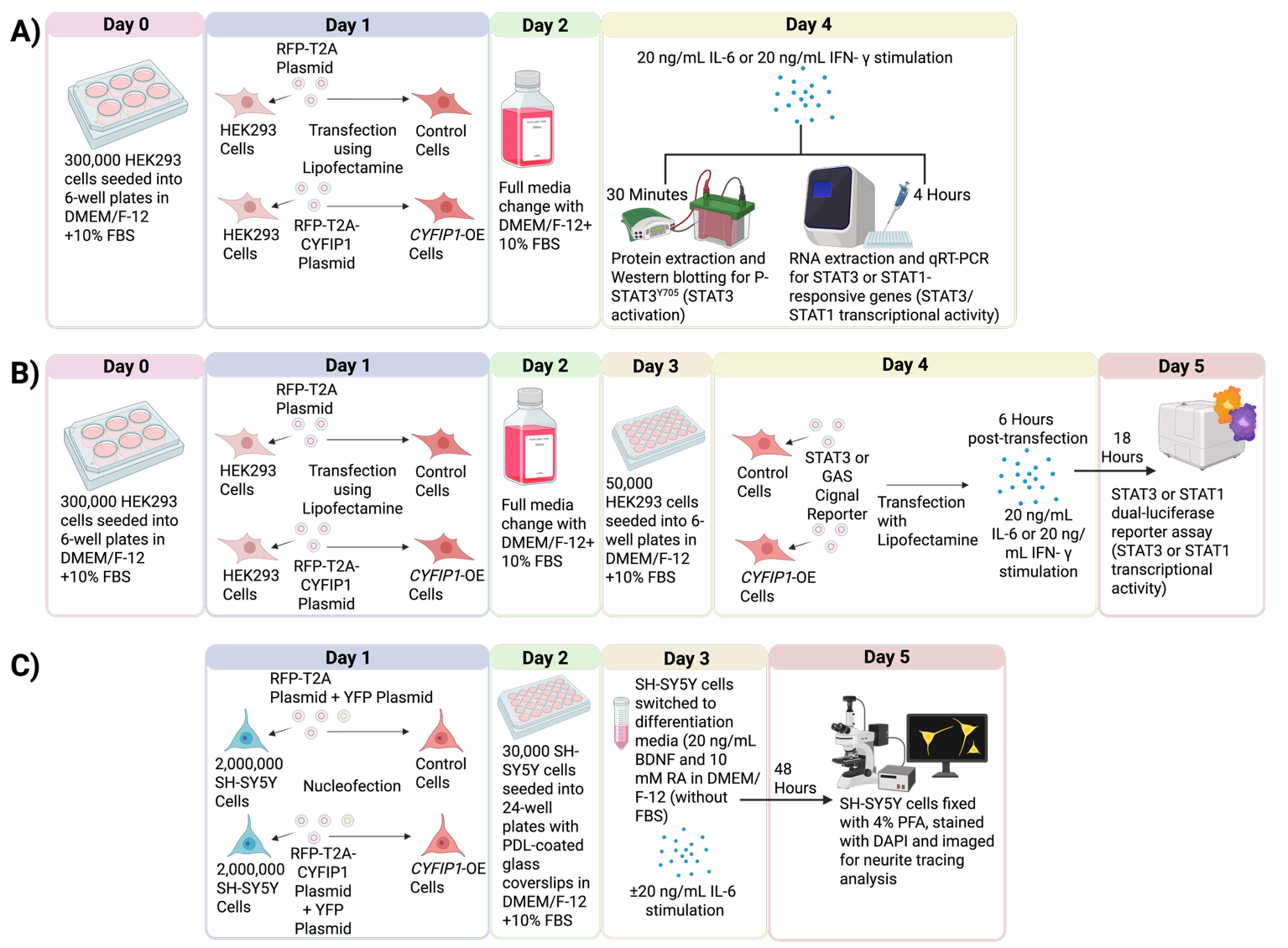


***Supplementary Figure 1 | Schematic overview of experimental methodologies. A)*** *Methodology for HEK-293 cell CYFIP1-OE Western blotting and qRT-PCR experiments to assess STAT3 activation (STAT3^Y705^ phosphorylation) and STAT3/STAT1 activity (STAT3/STAT1-responsive gene mRNA expression).* ***B)*** *Methodology for HEK-293 cell CYFIP1-OE dual luciferase reporter assay to assess STAT3 transcriptional activity.* ***C)*** *Methodology for SH-SY5Y CYFIP1-OE IL-6-stimulated neurite tracing experiments to assess neurite outgrowth. Created with Biorender.com.*


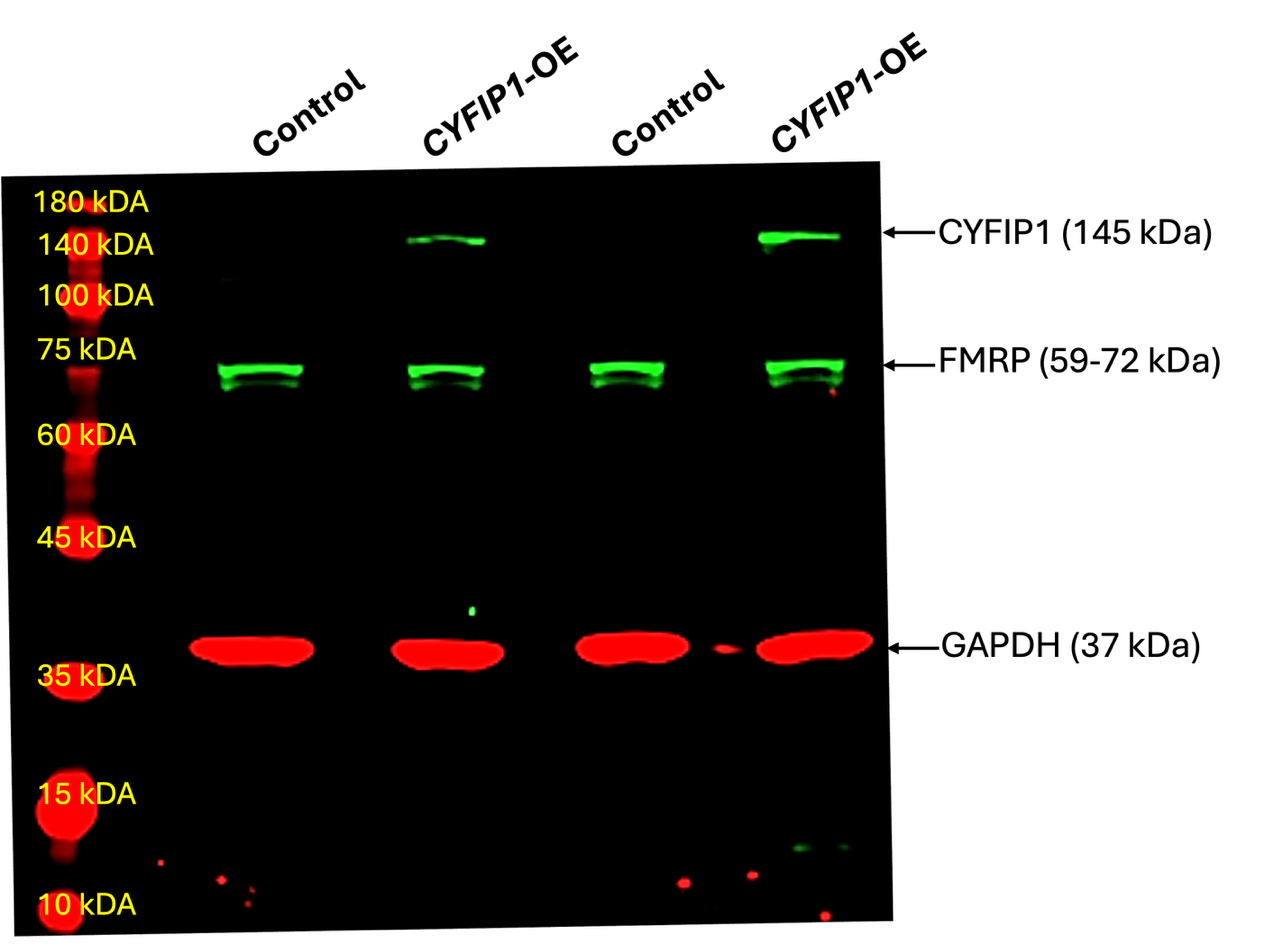


***Supplementary Figure 2 | Modelling CYFIP1-overexpression in HEK-293 cells.*** *Increased CYFIP1 protein expression in CYFIP1-OE cells 72 hours post-transfection confirmed via Western blotting. Membrane probed with: Rabbit Anti-CYFIP1 (#ab156016, Abcam), Rabbit Anti-FMRP (#13755-1-AP, Proteintech), Mouse Anti-GAPDH (#sc-47724, Santa Cruz Biotechnology), Goat Anti-Mouse DyLight™-680 (#35519, Invitrogen) and Goat Anti-Rabbit DyLight™-800 (#SA5-10036, Invitrogen).*


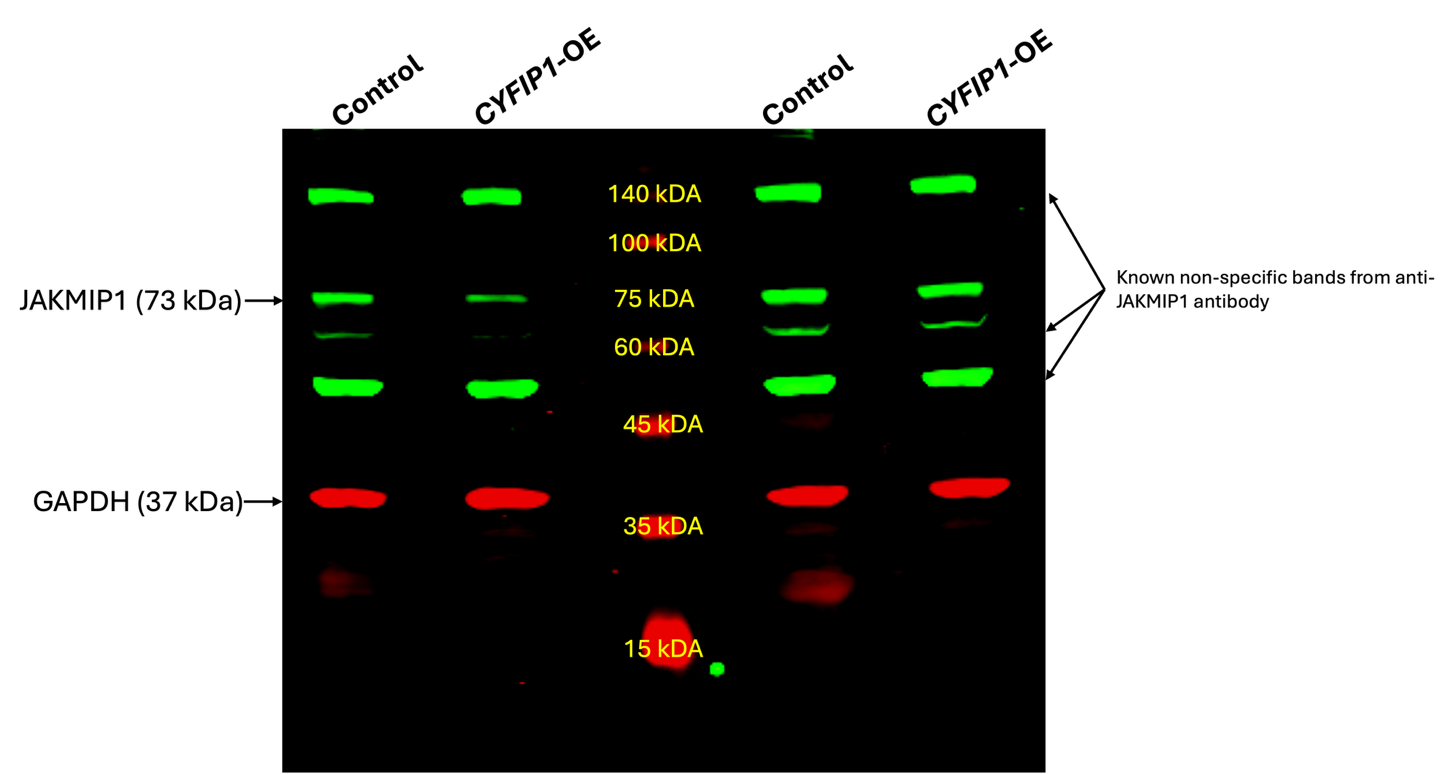


***Supplementary Figure 3 | CYFIP1-OE leads to reduced JAKMIP1 protein expression.*** *Western blotting of JAKMIP1 expression in Control and CYFIP1-OE cells. Membrane probed with: Rabbit Anti-JAKMIP1 (#13846-1-AP, Proteintech), Mouse Anti-GAPDH (#sc-47724, Santa Cruz Biotechnology), Goat Anti-Mouse DyLight™-680 (#35519, Invitrogen) and Goat Anti-Rabbit DyLight™-800 (#SA5-10036, Invitrogen). Known non-specific bands produced are indicated on the blot.*


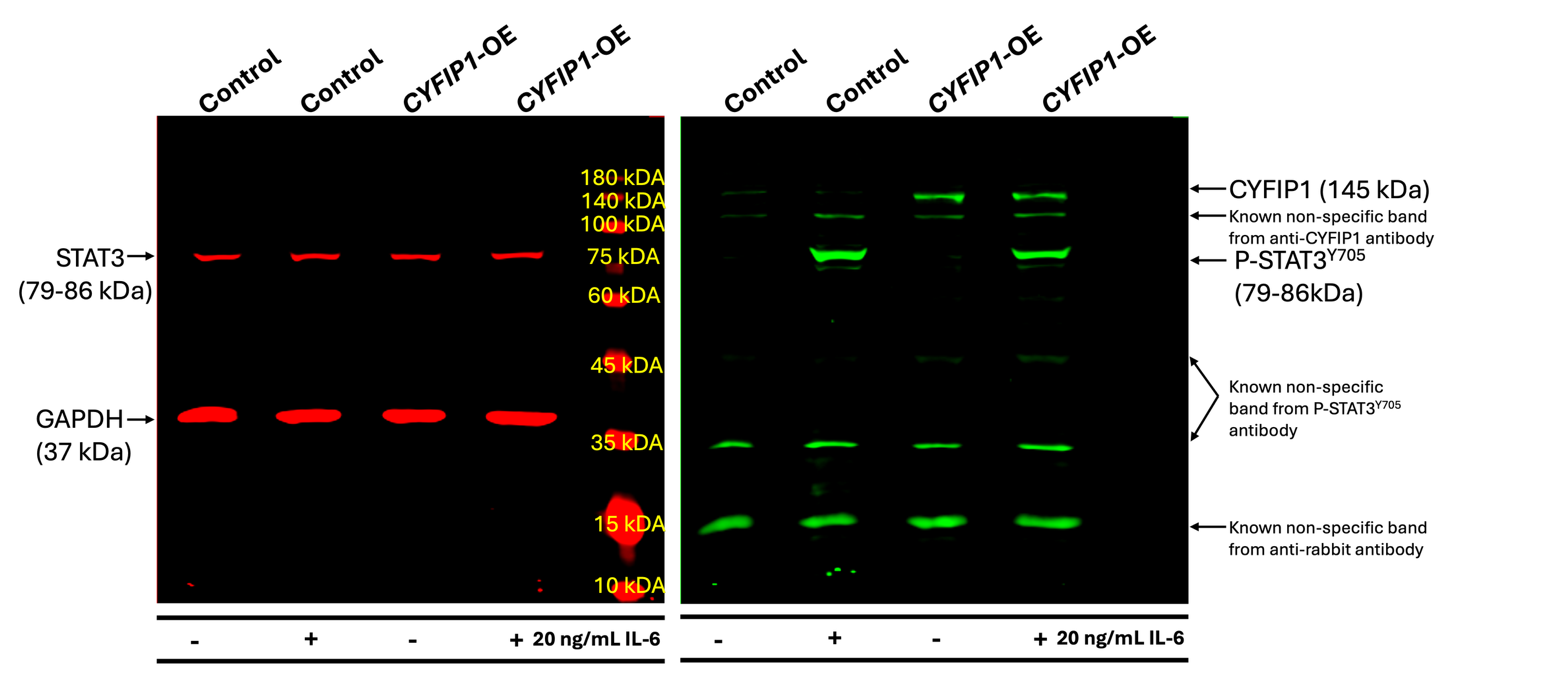


***Supplementary Figure 4 | CYFIP1-OE has no effect on STAT3 activation.*** *Western blotting of STAT3 expression and phosphorylation (P-STAT3^Y705^) in Control and CYFIP1-OE cells following 30-minute treatment with 0 ng/mL IL-6 or 20 ng/mL IL-6. Membrane probed with: Mouse Anti-STAT3 (#9139S, Cell Signaling Technology), Rabbit Anti-P-STAT3^Y705^ (#9131l, Cell Signaling Technology), Mouse Anti-GAPDH (#sc-47724, Santa Cruz Biotechnology), Goat Anti-Mouse DyLight™-680 (#35519, Invitrogen) and Goat Anti-Rabbit DyLight™-800 (#SA5-10036, Invitrogen). Known non-specific bands are indicated on the blot.*
